# Topical Exposure to *Nemopilema nomurai* Venom Triggers Oedematogenic Effects: Enzymatic Contribution and Identification of Venom Metalloproteinase

**DOI:** 10.3390/toxins13010044

**Published:** 2021-01-08

**Authors:** Yang Yue, Huahua Yu, Rongfeng Li, Pengcheng Li

**Affiliations:** 1 Key Laboratory of Experimental Marine Biology, Institute of Oceanology, Center for Ocean Mega-Science, Chinese Academy of Sciences, No. 7 Nanhai Road, Qingdao 266071, China; yueyang@qdio.ac.cn (Y.Y.); rongfengli@qdio.ac.cn (R.L.); 2 Laboratory for Marine Drugs and Biological Products, Qingdao National Laboratory for Marine Science and Technology, No. 1 Wenhai Road, Qingdao 266237, China

**Keywords:** jellyfish venom, metalloproteinase, oedema, vascular permeability, type IV collagen

## Abstract

Scyphozoan envenomation is featured as severe cutaneous damages due to the toxic effects of venom components released by the stinging nematocysts of a scyphozoan. However, the oedematogenic property and mechanism of scyphozoan venoms remain uninvestigated. Here, we present the oedematogenic properties of the nematocyst venom from *Nemopilema nomurai* (NnNV), a giant stinging scyphozoan in China, for the first time, using in vivo and in vitro models with class-specific inhibitors. NnNV was able to induce remarkable oedematogenic effects, including induction of significant oedema in the footpad and thigh of mouse, and increase in vascular permeability in the dorsal skin and kidney. Moreover, batimastat, a specific metalloproteinase inhibitor, could significantly reduce the Evan’s blue leakage in the damaged organs and attenuate paw oedema after 12 h, but exerted no influence on NnNV-induced thigh oedema. These observations suggested a considerable contribution of NnNV metalloproteinase-like components to the increased vasopermeability, and the participation was strongly suggested to be mediated by destroying the integrity of the vascular basement membrane. Moreover, partial isolation combined LC-MS/MS profiling led to identification of the protein species Nn65 with remarkable metalloproteinase activity. This study contributes to the understanding of the effector components underlying the cutaneous damages induced by scyphozoan stings.

## 1. Introduction

Jellyfish belong to the phylum Cnidaria, which are considered to be one of the most represented venomous taxa in the ocean, with 2% venomous species out of 11,000 known cnidarians [1,2]. Cubozoan, scyphozoan and hydrozoan of Medusozoa are all venomous creatures armed with formidable stinging organelles (i.e., nematocysts), of which cubozoan is thought to be the most dangerous and fatal to swimmers [3]. Jellyfish stings cause hospitalizations and death events worldwide, and therefore pose a great threat to the public health. It was estimated that about 150 million jellyfish stings occur in the world every year. Specifically, jellyfish sting is a consequence of direct contact with discharged nematocysts, however, the symptoms may vary depending on various factors, such as jellyfish species, envenomed area and toxicity potency.

*Nemopilema nomurai* is a stinging scyphozoan widely distributed in Chinese coastal waters [4]. The scyphozoan *N. nomurai* is generally considered to be less toxic than some cubozoan species, such as *Chironex fleckeri* and *Carukia barneis*. However, its toxicity is empirically higher than another venomous scyphozoan in China, *Cyanea nozakii*, according to clinical observations. In most cases, *N. nomurai* stings induce mild to moderate topical symptoms including redness, oedema, itch and immediate pain. Similar envenoming symptoms were also observed in other scyphozoan stings such as *Rhopilema nomadica* and *Rhizostoma pulmo* [5,6]. These topical cutaneous symptoms are often inflammatory and allergy, and termed as jellyfish contact dermatitis by physicians. Fundamentally, jellyfish dermatitis is the result of the combined effects of various venom compositions stored in the nematocysts. Recent years witnessed a great advance in deciphering the highly complex venom components by omics- and isolation-based approaches [7,8,9,10]. Unfortunately, the connection between cutaneous inflammatory reactions and the underlying material basis is scarcely explored.

Nematocyst venoms from *N. nomurai* exhibit potent lethality [11], hemolysis [12], cardiotoxicity [13], cytotoxicity [14], antioxidant and insecticidal activity [15,16]. Moreover, *N. nomurai* venom also possesses significant biochemical properties, such as enzymatic activities [17,18]. However, none of the biological and biochemical properties can explain well the cutaneous inflammatory reactions induced by *N. nomurai* stings. In our previous studies, we found *N. nomurai* venom exerted profound metalloproteinase and phospholipase-like activities [19,20]; whether these enzymatic components were involved in the topical envenomed cutaneous tissues is still elusive. In the present study, oedematogenic properties of *N. nomurai* nematocyst venom were firstly characterized by in vivo and in vitro models, and the connections between the oedematogenic property and the enzymatic activities were revealed using two specific enzyme inhibitors. This study contributes to the understanding of the toxicity and the effector compositions of *N. nomurai* nematocyst venom and provides an important reference for establishing the therapy of jellyfish dermatitis.

## 2. Results

### 2.1. Effects of Inhibitors of NnNV Components on Lethality and Oedema

**Figure 1:**
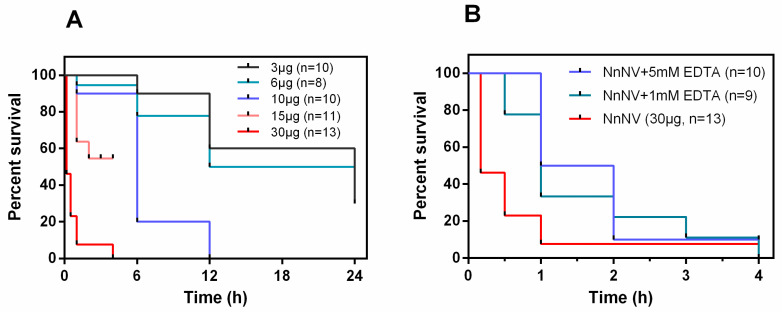
(**A**) Effects of varying amounts of NnNV on lethal activity to juvenile grass carp *Ctenopharyngodon idellus*. After 4 h of incubation with 15 µg of NnNV, five *C. idellus* died and the other six juvenile grass carp rolled over on their sides and lost their swimming ability; their survival rates over the next 20 h were not recorded by accident. (**B**) Effects of metalloproteinase inhibitor EDTA on the lethal activity of NnNV to juvenile grass carp. NnNV, *Nemopilema nomurai* nematocyst venom; EDTA, ethylenediaminetetraacetic acid; mM, mmol/L.

To test the toxic potency of the extracted nematocyst venom, the lethal activity was assayed. As illustrated in Figure 1A, 3–30 µg of NnNV exhibited potent lethal activity against juvenile grass carp *Ctenopharyngodon idellus*. High dosage of NnNV (30 µg) resulted in almost 100% death of *C. idellus* within 4 h, and most individuals immediately died within 1 h. The survival rates of *C. idellus* under low dosage of NnNV (3–6 µg) were determined to be 30–38% within 24 h, which were significantly lower than those of *C. idellus* preincubated by high doses of NnNV. Figure 1B shows the influence of ethylenediaminetetraacetic acid (EDTA) on the lethality of NnNV. EDTA at 1 mM significantly improved the survival rates from 7.7% (0.5 h) and 23.1% (1 h) to 77.8% (0.5 h) and 33.3% (1 h), respectively. Moreover, high doses of EDTA (5 mM) significantly reduced acute death induced by NnNV, with the survival rate at 1 h reaching 50.0%. These observations indicated that EDTA could slow down the death rate of *C. idellus* at 0.5–2 h. However, EDTA did not change the mortality of *C. idellus* within 4 h, as almost all *C. idellus* individuals died in the presence of 1 mM or 5 mM of EDTA.

**Figure 2:**
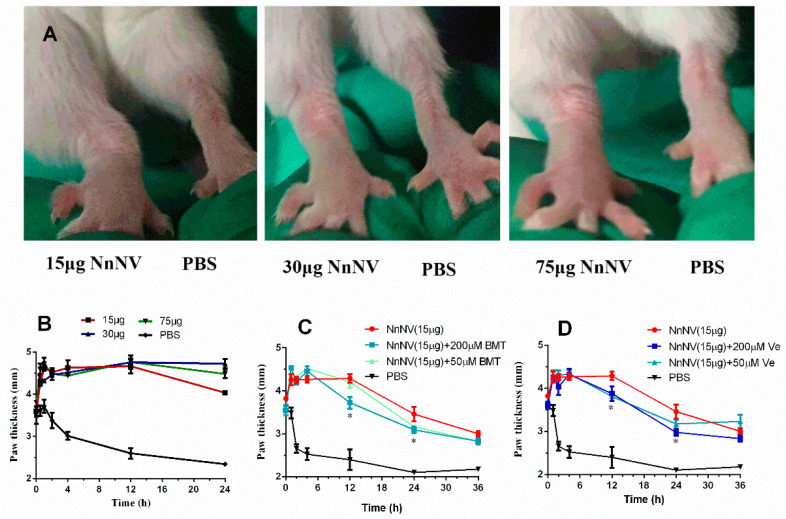
(**A**) Photos of paw oedema after injection of varying amounts of NnNV into the right hind paw of experimental animals at the time point of 4 h. (**B**) Oedematogenic activity of NnNV. Oedematogenic activity was expressed as paw thickness (mm), with measurement performed within 24 h. Inhibitory effects of batimastat (**C**) and varespladib (**D**) to the oedematogenic activity of NnNV within 36 h. Figure 2A–B and Figure 2C–D were from two independently performed experiments. NnNV, *Nemopilema nomurai* nematocyst venom; BMT, batimastat; Ve, varespladib; PBS, phosphate-buffered saline; μM, μmol/L. * *p* < 0.05 was considered as statistically significant.

The oedematogenic properties of NnNV were assayed with different animal models in vivo. In the acute inflammation model, NnNV was used as an acute proinflammatory agent to induce paw oedema. As shown in Figure 2A,B, intraplantar injection of 15–75 µg of NnNV/paw remarkably increased the paw oedema within 0.5–1 h, compared to the contralateral paw injected with saline. Moreover, the paw oedema induced by NnNV was longlasting within 24 h. To determine the possible role of NnNV components in the inflammatory oedema, two specific enzyme inhibitors, batimastat and verespladib, were used in this study. Batimastat was found to fail to inhibit the increase of paw oedema within 4 h at the concentrations of 50 and 200 μM (Figure 2C). However, the paw oedema induced by NnNV pretreated with 200 μM of batimastat was significantly lower than that of the NnNV control at 12 h and 24 h, respectively. A similar phenomenon was also observed in mice preincubated with 50 µM or 200 µM of varespladib (Figure 2D).

**Figure 3:**
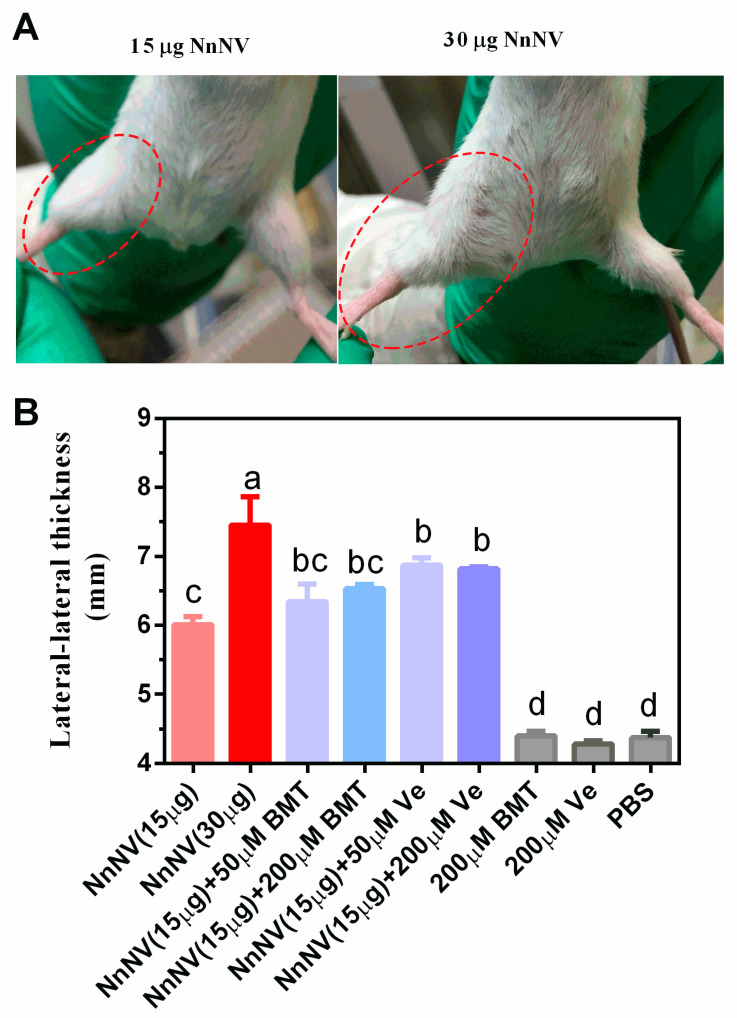
(**A**) Photos of thigh oedema induced by NnNV. The experiment animals received an intramuscular injection of 15–30 μg of NnNV into the right thigh (red dotted line). (**B**) Influence of batimastat and varespladib on NnNV-induced thigh oedema. NnNV, *Nemopilema nomurai* nematocyst venom; BMT, batimastat; Ve, varespladib; PBS, phosphate-buffered saline; μM, μmol/L. Statistical differences among different groups are indicated with different letters a–d (*p* < 0.05) following Duncan’s multiple comparison test.

The mouse thigh oedema was induced by intramuscular injection of NnNV and the degree of oedema was characterized as thigh thickness. As showed in Figure 3, 15 µg and 30 µg of NnNV could significantly increase thigh oedema within 24 h in a dose-dependent manner (*p* < 0.05). However, intramuscular injection of batimastat or verespladib did not result in reduction of thigh oedema compared to the venom control group (15 µg of NnNV).

### 2.2. Induction of Vascular Permeability and Proteolysis of Vascular Basement Membrane Components by NnNV

**Figure 4:**
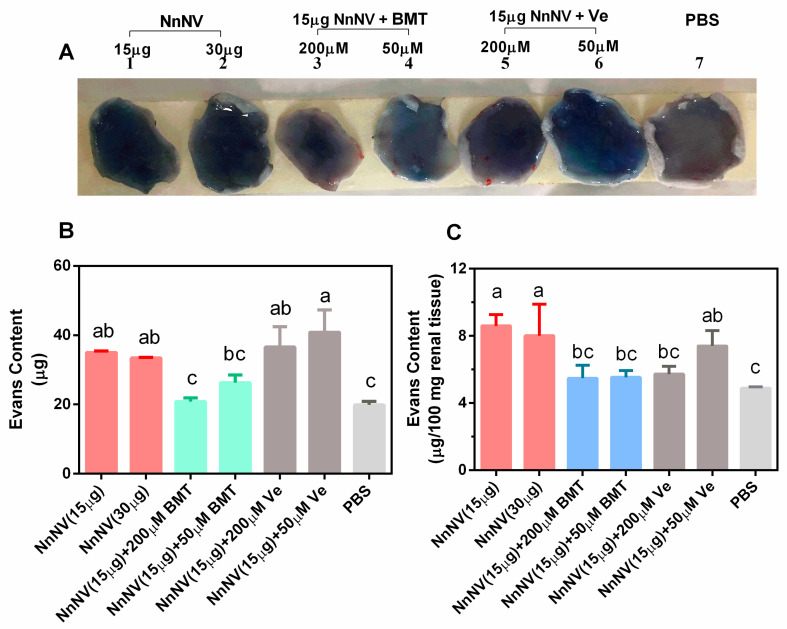
(**A**) Photos of vasopermeability changes induced by NnNV in mouse dorsal skin with or without inhibitors. (**B**) Quantitative determinations of the inhibition of batimastat or varespladib on the vascular permeability in the dorsal skin (**B**) and kidney (**C**). NnNV, *Nemopilema nomurai* nematocyst venom; BMT, batimastat; Ve, varespladib; PBS, phosphate-buffered saline; μM, μmol/L. Statistical differences among different groups are indicated with different letters a–c (*p* < 0.05) following Duncan’s multiple comparison test.

Figure 4 shows the dye Evan’s blue leakage in the dorsal skin and kidney induced by NnNV. Visual examination of the dorsal skin tissues indicated that NnNV greatly enhanced the dye leakage compared to the phosphate-buffered saline control (PBS, Figure 4A). Further quantitative analysis affirmed the marked increase of Evan’s blue leakage in the dorsal skin tissues and kidneys of the NnNV-treated mice (*p* < 0.05, Figure 4B,C). Visual reduction of Evan’s blue leakage in the dorsal skin tissues was observed in batimastat-treated mice, however, the leakage reduction was significant only in mice treated with high doses of batimastat (200 µM) (*p* < 0.05). Figure 4C also revealed that the dye Evan’s blue leakage in the kidney significantly decreased in batimastat- and varespladib-treated mice (*p* < 0.05). These results indicated that batimastat (50 µM and 200 µM) and varespladib (200 µM) played a microvascular protective role in the dorsal skin and kidney in NnNV-envenomed mice.

**Figure 5:**
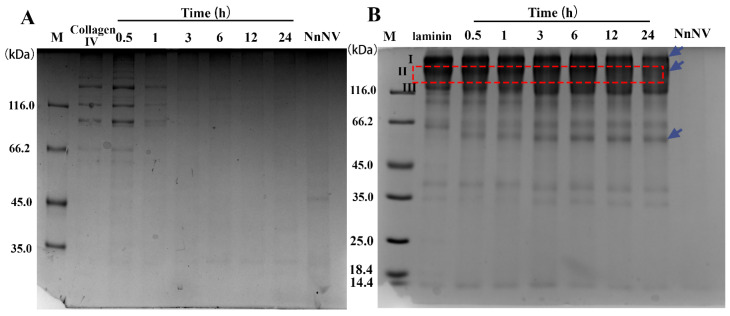
Proteolysis of vascular basement membrane components Type IV collagen (**A**) and laminin (**B**) by NnNV within 24 h. NnNV, *Nemopilema nomurai* nematocyst venom; M, Marker; NnNV, venom control; Collagen Type IV and laminin serve substrate control.

To investigate the proteolytic effects of NnNV on the vascular basement membrane, Type IV collagen and laminin were included in this study. NnNV was able to degrade type IV collagen with high efficiency; all chains of the type IV collagen almost completely disappeared after 3 h of incubation (Figure 5A). Moreover, hydrolysis of laminin by NnNV were also observed after 0.5 h of incubation with a new band being produced at ~60 kDa. After 24 h of incubation, the band intensity of laminin subunit I and II decreased significantly (Figure 5B), suggesting that the integrity of laminin was destroyed by NnNV.

### 2.3. Isolation and Identification of Enzymatic Components from NnNV

**Figure 6:**
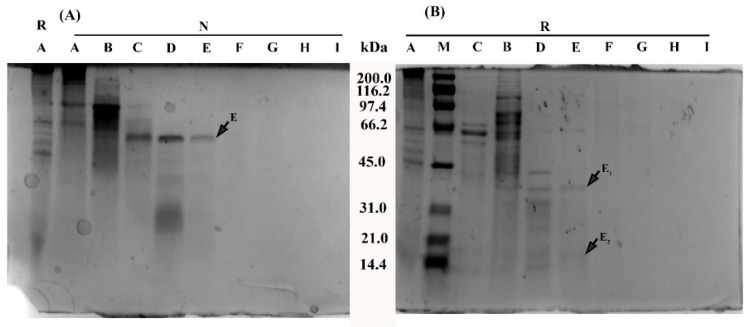
SDS-PAGE profiles of NnNV and its fractions peak A–I under nonreducing (**A**) and reducing (**B**) conditions. M, Marker. The protein bands in Fraction E under reducing (R) and nonreducing (N) conditions were excised into three pieces of gel strips, as indicated by arrows, and subjected to in-gel digestion for identification.

To identify the possible enzymatic components responsible for the proteolytic effects of NnNV, bioassay guided separation was conducted by following the enzymatic activities of NnNV. About 15 mg of NnNV were subject to fractionation with HiPrep 26/60 Sephacryl S-200 column (Figure S1). Ten fractions Peak A–J were obtained, and were then subject to ultrafiltration using a membrane with a cut-off value of 3000 Da. Figure 6 showed the SDS-PAGE profiles of fractions Peak A–I under reducing (R) and nonreducing (N) conditions. After isolation, major proteinaceous components in NnNV were fractionated into Peak A–E, as no visible protein bands were observed in the SDS-PAGE of Peak F–I. The observations were consistent with the results of the protein contents of fractions Peak A–J (Table S1). Under nonreducing conditions, only one band was visible for Peak E, and its molecular weight was about 65 kDa. Moreover, reducing SDS-PAGE revealed two new bands at 14.4–45.0 kDa, indicating that the protein at 65 kDa may consist of two subunits connected by disulfide bonds.

**Figure 7:**
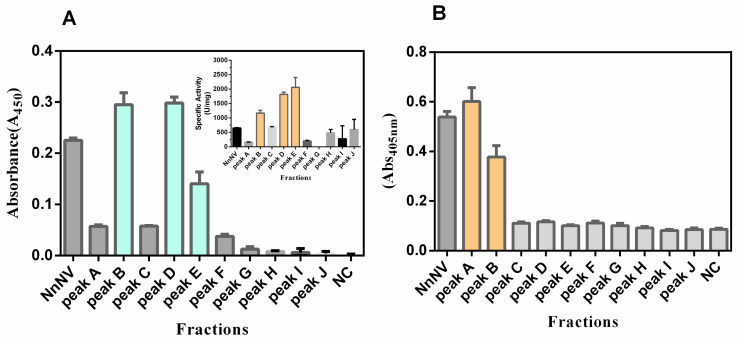
Metalloproteinase activity (**A**) and phospholipase activity (**B**) of NnNV and fractions A–J. The insert shows the specific activity (U/mg). All results were repeated four times and expressed as mean ± S.E.M (n = 4). NnNV, *Nemopilema nomurai* nematocyst venom; NC, negative control (phosphate-buffered saline).

The metalloproteinase activity and phospholipase activity of fractions Peak A–J were presented in Figure 7. Results suggested that Peak B, D and E possessed the most significant metalloproteinase activity on azocasein, and Peak E exhibited the highest specific activity, with a value of 1818.0 ± 36.5 U/mg (mean ± S.E.M, *n* = 4, Figure 7A insert). Figure 7B showed that NnNV components exerting significant phospholipase activity on the substrate 4-nitro-3-octanoyloxybenzoic acid (NOBA) were largely pooled into fractions Peak A and B. Of note, Peak B could hydrolyze both azocasein and NOBA, while Peak D and E only showed significant proteolytic effects on azocasein. These results indicated that NnNV proteases were largely fractionated into Peak D and E.

Considering the SDS-PAGE profile of Peak E, the band at ~65 kDa and its two subunits under reducing conditions were subject to LC-MS/MS profiling and identification using UniProt animal venom and toxins database. As presented in Table 1, the top hits for Nn65 were TALE class homeobox transcription factor Meis, Pp4 and retinoid X receptor. No known proteases were completely matched with high similarity.

## 3. Discussion

Scyphozoan envenomation is generally characterized by mild to moderate local lesions, in rare cases inducing severe skin damage and systemic symptoms. The topical cutaneous lesions are not life-threating, but they seriously impact the patient’s daily activities. Effective therapies improving the topical damages are still elusive due to lack of understanding of the envenoming mechanisms. Therefore, deciphering the effector molecules underlying the sting-induced topical damages is of great interest for the scientific community. In the present study, the oedematogenic properties of *N. nomurai* nematocyst venoms were firstly reported, and *N. nomurai* venom metalloproteinases were suggested to be involved in oedema formation by a relevant mechanism of vascular basement membrane degradation.

Oedema is a typical sign of local inflammation, and its formation is often correlated with exudation of plasma proteins and accumulation of inflammatory cells, such as neutrophils and macrophages [21]. Generally, an acute inflammatory response may develop by injection of an irritant substances (i.e., carrageenan) in one hind footpad of experimental animals. In the carrageenan-induced acute inflammation model, the animals exhibited marked oedema and hyperalgesia at the injection site, which resulted from the release of various inflammatory mediators, for example, histamine, bradykinin and nitric oxide [22]. Oedema induced by venomous animal bites or stings were extensively reported in animal models and clinical observations [23]. Case reports on jellyfish stings from scyphozoans [24], cubozoans [25] and hydrozoans [26] suggested a prevalent occurrence of swelling in the envenomed area. However, the oedematogenic property of jellyfish venoms was still uninvestigated. Nematocyst venom from scyphozoan *N. nomurai* was able to induce significant paw oedema and thigh oedema, which indicates that jellyfish venom may function as a proinflammatory material. As we know, the formation of oedema is a result of extravasation of plasma components to the affected tissue. The present study affirmed that *N. nomurai* nematocyst venom significantly augmented microvascular permeability, resulting in an increase of Evan’s blue leakage in the dorsal skin and kidneys (Figure 4). The change in vasopermeability may be attributed to a direct or indirect effect of jellyfish venom on capillaries. Direct hydrolysis of the vascular basement membrane by venoms from various snakes was reported [27,28,29]. Studies indicated that type IV collagen was a key target for snake venom metalloproteinases to break the integrity of microvascular wall [30]. Remarkable degradation of type IV collagen was also observed for *N. nomurai* nematocyst venom, and the proteolytic effect was greatly stronger than that of another basement membrane component, laminin. This study supported direct hydrolysis of structural basement membrane components by jellyfish venom, resulting in vasopermeability changes.

Participation of enzymatic components of snake venoms in the pathogenesis of oedema was validated in many studies [31,32]. Snake venom metalloproteinases and phospholipase A_2_ are thought to play vital roles in the formation of oedema [33]. Jellyfish venom is a mixture of various proteinaceous components including many enzymatic components, such as proteases, lipases, and hyaluronidases [34]. Jellyfish nematocyst venom metalloproteinases (JnVMPs) were suggested to include a group of catalytically and biologically active proteases, according to our previous studies [19,20]. However, whether JnVMPs contribute to oedema is unknown. Batimastat, a specific metalloproteinase inhibitor, was able to effectively inhibit the catalytic activity of *N. nomurai* nematocyst venom. In the present study, batimastat was found to fail to inhibit thigh oedema and acute inflammatory paw oedema within 4 h, but significantly reduced paw swelling in the following 12–24 h. The phenomenon indicated that JnVMPs may not act as an acute proinflammatory agents but play roles in the persistence of inflammation. A similar phenomenon was observed in the varespladib-treated group. The participation of JnVMPs in oedema is primarily attributed to its effect on vascular permeability, since batimastat (200 µM) induced marked reduction of Evan’s blue dye extravasation in the dorsal skin and kidney. No effect was observed for varespladib on dorsal vascular permeability, suggesting no participation of phospholipase in the vasopermeability change induced by *N. nomurai* venom. In the clinical therapies for curing scyphozoan envenomations, antihistaminics were routinely used to attenuate the skin lesions, including oedema [24,35]. The present findings rationalize the administration of antihistaminics in scyphozoan stings, since JnVMPs significantly increased the vascular permeability. 

Although detected in the proteome and the nematocyst extracts, JnVMPs are still a class of metalloproteinases that are not well understood. *N. nomurai* nematocyst venom metalloproteinases (NnVMPs) consist of at least nine stable and proteolytically active proteases at 28–46 kDa (I), 57–83 kDa (II) and 139 kDa (III) [36]. This study represents the first attempt to isolate and identify these NnVMPs. Following fractionation by Sephacryl S-200 column, NnVMPs were exclusively detected in Fractions D-E, which were absent in phospholipase activity. Fraction E exhibits one single band at about 65 kDa, termed as Nn65, under nonreducing SDS-PAGE and possesses the highest specific activity on azocasein. Tandem LC-MS profiling shows that TALE class homeobox transcription factor Meis (~63 kDa) was the top hit for Nn65, however, as far as we know, no clues indicate that the TALE class homeobox transcription factor Meis functions as a protease. Therefore, the metalloproteinase activity of fraction E was not well explained by the MS/MS matching results. Future efforts should be addressed to decipher the physiochemical nature of JnVMPs and understand their toxicological relevance.

It is worth mentioning that the present study is limited to roughly presenting the oedematogenic properties of NnNV in vivo. We used 15–75 µg of NnNV to trigger the oedematogenic effect, but we did not determine the minimum dosage. Only in vitro hydrolysis of vascular basement membrane proteins was observed, and the degradation was not validated in vivo models, which may better reflect the oedematogenic effect of jellyfish venom. Moreover, we were also limited to only LC-MS/MS identification of peak E; other fractions with significant metalloproteinase activities were not examined.

In conclusion, the present study investigated the oedematogenic and lethal properties of *N. nomurai* nematocyst venom with class-specific inhibitors, revealing the contribution of NnVMPs to the increased vasopermeability through a mechanism of direct degrading basement membrane components. In addition, fractionation-based LC-MS/MS profiling identified a protein species with remarkable metalloproteinase activity. The current study sheds new light on the cutaneous damages induced by scyphozoan envenomation.

## 4. Materials and Methods 

### 4.1. Chemical and Regents

Azocasein was purchased from Sigma Chemical Co. (St. Louis, MO, USA). Batimastat (BMT) and 4-nitro-3-octanoyloxybenzoic acid (NOBA) were from Abcam (Shanghai, China). All other regents used were of analytical grade.

### 4.2. Sample Collection and Venom Preparation 

Specimens *N. nomurai* were collected from the coast of Qingdao in August of 2015–2016. Tentacles of *N. nomurai* were manually excised and stored at −80 °C. For venom extraction, nematocysts were isolated as described previously [19]. Briefly, the frozen tentacles were placed at 4 °C for autolysis for several days. Then, the tissue debris were removed by a 200-mesh plankton net. The filtrate was centrifuged at 1000× *g* for 15 min at 4 °C, and the sediments (nematocysts) were used for venom extraction. The nematocysts were suspended in 20 mM of phosphate buffer (pH 7.4) containing 150 mM of NaCl, and were subjected to 400–600 W for 90 cycles using Ultraturrax (JY92-II, Scientz, Ningbo City, China). Each cycle consisted of 10 s sonication and 15 s interval rest on ice. The extracts were combined and centrifuged at 20,000× *g* for 15 min at 4 °C and labeled as NnNV (*Nemopilema nomurai* Nematocyst Venom) for convenience. Protein concentration was determined using a lowry protein assay kit (DingGuo ChangSheng Biotechnology Co. Ltd., Beijing, China), according to the manufacture’s introductions.

### 4.3. Venom Lethality

The lethal effect of NnNV was evaluated using juvenile grass carp *Ctenopharyngodon idellus*. The fish were purchased from a market and housed in the laboratory for a week before experiments. Individuals possessing too large or small body size were manually excluded from the study. Varying doses of venom (0.3–30 μg/fish) in a volume of 20 µL were injected around the tail of the grass carp. In each group, 8–13 fishes were included. To test the effect of EDTA on NnNV-induced lethality, NnNV (30 μg) was preincubated for 30 min with EDTA (1 mM, 5 mM, final concentrations), then injected into the grass carp. Deaths were recorded within 24 h and survival rates were calculated.

### 4.4. Animal Maintenance

All animal tests were performed with Institute of Cancer Research (ICR) mice, which were purchased from Qingdao Pharmaceutical Inspection Institute. The animals were kept in an animal care facility under controlled temperature and normal day and night cycles and were acclimatized for one week in laboratory conditions before carrying out the experimental study. The ICR mice had free access to food and water. All efforts were made to minimize animal suffering and to reduce the number of animals used, according to Guide for the Care and Use of Laboratory Animals (8th version, 2011). All experimental procedures were approved by the Committee on the Ethics of Animal Experiments of the Institute of Oceanology, Chinese Academy of Sciences (Protocol code: No. IOCAS/KLEMB/20170301; Date: 2017/03/17). 

### 4.5. Evaluation of Jellyfish Venom-Induced Oedema

The oedematogenic property of NnNV was determined by subplantar injection of jellyfish nematocyst venom into the hind paw of mice, according to a modified method [30]. To minimize the numbers of animals, the ICR mice were randomly grouped into 4 groups of 3 mice, and were allowed to adapt to the environment before study. Totals of 15–75 µg/paw of NnNV dissolved in 50 µL of PBS were subplantarly injected into the hind paw of the ICR mice, with the contralateral paw injected with isotonic PBS. Then, paw oedema was measured using micrometer calipers at 0, 0.5, 1, 2, 4, 12, 24 h following NnNV injection. The thickness of the hindpaw were recorded to reflect the degree of the oedema induced by NnNV. To investigate the effects of varespladib and batimastat on NnNV-induced oedema, NnNV was pretreated for 30 min with 50 mM and 200 mM of batimastat or varespladib at 37 °C, then the oedema was measured as described above.

### 4.6. Thigh Oedema Assay

Thigh oedema induced by NnNV was used to evaluate the influence of NnNV on ICR mice muscle, according to the previously reported method [37]. Briefly, the posterior aspect of the right thigh received an intramuscular injection of 15–30 µg of NnNV. Then, the oedema was measured with micrometer calipers after 24 h of injection. The lateral–lateral diameter of the mice tights was recorded. Similarly, to investigate the effects of varespladib and batimastat on NnNV-induced thigh oedema, NnNV was pretreated for 30 min with 50 mM and 200 mM of batimastat or varespladib at 37 °C. Then, thigh oedema was measured as described above. The animals that received PBS, batimastat (200 µM) or varespladib (200 µM) alone were used as negative controls, respectively.

### 4.7. Vascular Permeability Assay

Effects of NnNV on vascular permeability were examined according to a previously reported method [38]. Briefly, 5% (*w*/*v*) of Evan’s blue dye solution was freshly prepared in phosphate-buffered saline (PBS) and filtered before use. The ICR mice were intravenously injected with the Evan’s blue (5 mL/kg body weight). Sixty minutes later, groups of 3 mice received an intradermal injection of 15–30 µg of NnNV dissolved in 50 µL of PBS or 50 µL of PBS alone at the shaved dorsal skin of the animal. After 12 h, the mice were anesthetized by Zoletil^®^ 50, then the dorsal skin and the kidney were removed. The injected skin tissues were punched out and photographed to visualize the Evan’s blue leakage. To quantify the dye leakage, the back skin and kidney were soaked in 2 mL of acetone–H_2_O solution (7:3, *v*/*v*), respectively, for 48 h at room temperature. The amount of Evan’s blue leakage was determined by measuring the absorbance at 620 nm according to a standard curve of Evan’s blue. Results were expressed in micrograms of Evan’s blue dye leakage (µg or µg/100 mg kidney). To investigate the effects of varespladib and batimastat on NnNV-induced change in vascular permeability, 15 µg of NnNV pretreated for 30 min with 50 mM and 200 mM of batimastat or varespladib at 37 °C was injected at the dorsal skin, then the dye leakage was measured, as described above.

### 4.8. Proteolysis of Vascular Basement Membrane Proteins by NnNV In Vitro

The proteolytic activity of NnNV on different substrates in vitro was determined according to the previously described method [29]. Type IV collagen and laminin (1 mg/mL) were dissolved in 50 mM Tris–HCl (pH 7.5) containing 150 mM NaCl, and were incubated with NnNV at ratios of 5:1 at 37 °C for 0.5, 1, 3, 6, 12, and 24 h. At each time point, 20 µL of reaction mixtures were mixed with equal volume of 2× sample buffer (containing β-mercaptoethanol). Electrophoresis was performed using 12% polyacrylamide gels under reducing conditions and the gels were visualized after staining with 0.25% Coomassie Brilliant Blue R-250.

### 4.9. Fractionation of NnNV by Size Exclusion Gel

15 mg of NnNV dissolved in 10 mL of 20 mM of phosphate buffer (pH 7.4) containing 150 mM of NaCl was applied to a Sephacryl S-200 column (110 cm × 2.5 cm, GE Life Sciences, Chicago, IL, USA) connected to an FPLC system AKTA Pure (GE Life Sciences, Chicago, IL, USA). The column was equilibrated and eluted with 20 mM Tris–HCl (pH 8.0) containing 100 mM NaCl. Fractions of 3 mL/tube were collected, the flow rate was set to be 36 mL/h and the elution was monitored at 280 nm and 214 nm. Fractions were combined according to the absorbance at 280 nm. The combined fractions Peak A-I were concentrated by ultrafiltration (MWCO 3 kDa, Millipore, Burlington, MA, USA) and were subject to protein concentration determination, SDS-PAGE profiling and characterizing the enzymatic activities.

### 4.10. SDS-PAGE Analysis

Electrophoresis was performed using 12% SDS-PAGE gel under reducing and nonreducing conditions, according to a previously reported method [19]. Briefly, 20 µL of concentrated fractions were loaded into the sample lanes of the 12% SDS-PAGE gel, and electrophoresis was carried out at 120 V using Mini-PROTEAN Tetra apparatus (Bio-Rad, Hercules, CA, USA). Under nonreducing conditions, concentrated fractions were treated in the absence of heat (100 °C) and β-mercaptoethanol. Following electrophoresis, the gels were visualized by staining with 0.25% Coomassie Brilliant Blue R-250.

### 4.11. Proteolytic Activity on Azocasein

Proteolytic activity of NnNV was measured according to our previously reported method [19]. Briefly, 20 μL of fractions Peak A-J were added to 80 μL of azocasein solution dissolved in 50 mM Tris–HCl, 100 mM NaCl and 5 mM CaCl_2_, pH 8.8, at a concentration of 5 mg/mL. The reaction mixtures were placed at 37 °C for 90 min. Then, the reactions were terminated by addition of 200 μL of 0.5 M trichloroacetic acid at room temperature. 30 min later, the mixtures were subjected to centrifugation at 10,000 g for 15 min. 150 μL of the supernatant was transferred out and equal volumes of 0.5 M NaOH were added. Then, the absorbance was measured at 450 nm using Infinite M100 plate reader (Tecan Group Ltd., Männedorf, Switzerland). One unit of activity was defined as an increase of 0.01 absorbance units at 450 nm. The experiments were repeated four times and the results were expressed as specific activity (U/mg).

### 4.12. Phospholipase-Like Activity on NOBA

Phospholipase-like activity was measured with substrate 4-nitro-3-octanoyloxybenzoic acid (NOBA), as described previously [19]. Briefly, 25 μL of NnNV or isolated fractions were added to 200 μL of 50 mM Tris–HCl, 5 mM CaCl_2_ and 100 mM NaCl, pH 8.0, in quadruplicate in a 96-well plate. The reaction was initiated by adding 25 μL of NOBA (1 mg/mL in acetonitrile). Following incubation for 60 min at 37 °C, the absorbance at 405 nm was recorded. Samples of 25 μL of 50 mM Tris-HCl buffer replacing the venom fractions served as the negative control. In addition, the absorbance readings at 405 nm raised by the venom fractions themselves were subtracted.

### 4.13. In-Gel Digestion and LC-MS/MS Identification of Fraction E

The putative protein Nn65 in fraction E (Figure 7) was subjected to LC-MS/MS identification. The protein band E (Figure 7A) under nonreducing conditions and bands E_1_ and E_2_ (Figure 7B) under reducing conditions were cut from the gel. Then, in-gel digestion was performed as follows. The bands were destained twice for 10 min with 50% acetonitrile and 25 mM ammonium bicarbonate, then dehydrated with 100% acetonitrile. Then, the bands were incubated with 10 mM dithiothreitol and 55 mM iodoacetamide, then dehydrated with neat acetonitrile and dried under reduced pressure. The samples were then digested with trypsin (1 µg/µL) diluted in 25 mM ammonium bicarbonate at 37 °C overnight. For LC-MS/MS, 10 µL of extracted tryptic peptides were submitted to a MicrOTOF-QII mass spectrometer (Bruker Daltonics) for LC-MS/MS analysis. The isolation was performed with a C18 reverse column (5 µm,150 Å, Eprogen) connected to prominence nano 2D HPLC system (shimazhu). Solvents A and B consisted of 0.1% *v*/*v* formic acid and 100% acetonitrile (containing 0.1% formic acid, *v*/*v*), respectively. The separation condition was set as follows: 0~4 min, 5% B; 5~30 min, 5~40% B; 31~35 min, 40~80% B. Eluted peptides were analyzed in positive ion mode and the capillary voltage was set at 1500 V. The full scan ranged from *m*/*z* 50 to 2000 Da and the 20 most intense peptide ions were automatically selected for tandem mass spectrometry using collision induced fragmentation in the linear ion trap. The Mascot search engine version 2.3.01 (Matrix Science, London, UK) was used for database searching against an online SWISS-PROT toxin database (http://www.uniprot.org/program/Toxins).

### 4.14. Statistical Analysis

Results were expressed as mean ± SEM. The significance of differences between the means of various experimental groups was analyzed by Analysis of Variance, followed by Duncan’s multiple comparisons using SPSS Statistics 22.0 software (IBM, Armonk, New York, NY, USA). * *p* < 0.05 was considered as statistically significant.

## Figures and Tables

**Table 1:** Identification of the putative metalloproteinase Nn65 in fraction E by MS/MS spectral matching using the SWISS-PROT toxin database.

Protein	Sequence	*M*_w_	Accession	Description	Score
Nn65 (E)	KPSGGMIHSPVGPGIR	795	E3UJT4_MNELE	TALE class homeobox transcription factor Meis OS = Mnemiopsis leidyi	83
	KDSENDVER	546	A0A1W6LRY4_9CNID	Pp4 OS = Cladonema pacificum	14
	LQIDVSEWGCLR	738	R9YI93_9CNID	Retinoid X receptor OS = Clytia hemisphaerica	13
Nn65 (E_2_)	KPSGGMIHSPVGPGIR	795	E3UJT4_MNELE	TALE class homeobox transcription factor Meis OS = Mnemiopsis leidyi	110
	MLAPTEPLLSK	600	E3UJW2_MNELE	SIX class homeobox transcription factor SIX59b OS = Mnemiopsis leidyi	15
	LQIDVSEWGCLR	738	R9YI93_9CNID	Retinoid X receptor OS = Clytia hemisphaerica	13

## Data Availability

The data presented in this study are available on request from the corresponding author. The data are not publicly available due to privacy.

## Supplementary Materials

The following are available online at https://www.mdpi.com/2072-6651/13/1/44/s1, Figure S1: Gel filtration chromatogram of NnNV fractionated by HiPrep 26/60 Sephacryl S-200 column. Fractions were collected and combined as labeled A–J. Table S1: Determination of the protein concentrations of Fractions Peak A–J.
