# Supplementary material for: Topical Exposure to Nemopilema nomurai Venom Triggers Oedematogenic Effects: Enzymatic Contribution and Identification of Venom Metalloproteinase
Source: Toxins (Basel). 2021 Jan 8;13(1):44. doi: 10.3390/toxins13010044 (PMC7826907; doi:10.3390/toxins13010044)
Supplement: Supplementary file 1 [file toxins-13-00044-s001.pdf]

Supplementary Materials: Topical Exposure to *Nemopilema nomurai* Venom Triggers Oedematogenic Effects: Enzymatic Contribution and Identification of Venom Metalloproteinase

Yang Yue, Huahua Yu, Rongfeng Li and Pengcheng Li

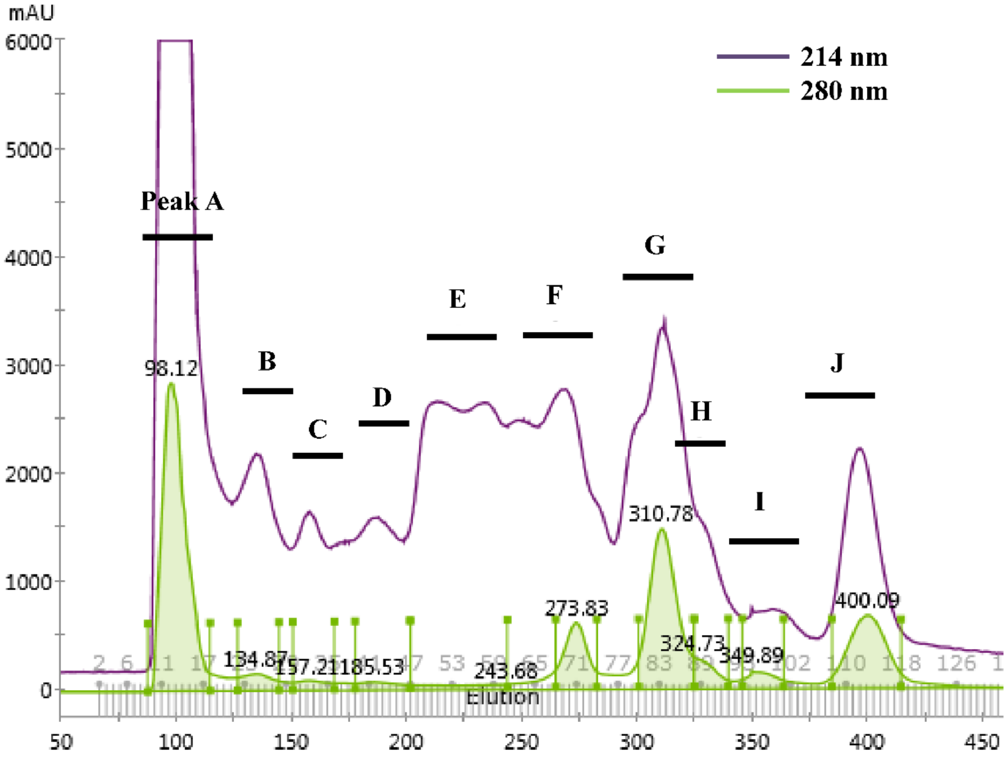

Figure S1. Gel filtration chromatogram of NnNV fractionated by HiPrep 26/60 Sephacryl S-200 column. Fractions were collected and combined as labeled A–J.

Table S1. Determination of the protein concentrations of Fractions Peak A–J.

Fractions	A	B	C	D	E	F	G	H	I	J
Concentration (mg/mL)	1.72	1.26	0.42	0.82	0.34	0.94	-	0.08	0.05	0.02
Standard curve	$Y = 0.0084 \times X + 0.002726$ ($R^2 = 0.9988$)									
